# `Whose Shoes?` Can an educational board game engage Ugandan men in pregnancy and childbirth?

**DOI:** 10.1186/s12884-018-1704-6

**Published:** 2018-03-27

**Authors:** Alice Norah Ladur, Edwin van Teijlingen, Vanora Hundley

**Affiliations:** Faculty of Health and Social Sciences, Bournemouth University, Royal London House, Christchurch Road, Bournemouth, BH1 3LT UK

**Keywords:** Safe motherhood, Utilisation, Facility-birth, Male involvement, Educational board games

## Abstract

**Background:**

Men can play a significant role in reducing maternal morbidity and mortality in low-income countries. Maternal health programmes are increasingly looking for innovative interventions to engage men to help improve health outcomes for pregnant women. Educational board games offer a unique approach to present health information where learning is reinforced through group discussions supporting peer-to-peer interactions.

**Methods:**

A qualitative study with men from Uganda currently living in the UK on their views of an educational board game. Men were purposively sampled to play a board game and participate in a focus group discussion. The pilot study explored perceptions on whether a board game was relevant as a health promotional tool in maternal health prior to implementation in Uganda.

**Results:**

The results of the pilot study were promising; participants reported the use of visual aids and messages were easy to understand and enhanced change in perspective. Men in this study were receptive on the use of board games as a health promotional tool and recommended its use in rural Uganda.

**Conclusions:**

This study provides preliminary data on the relevancy and efficacy of using board games in maternal health. Key messages from the focus group appeared to be that the board game is more than acceptable to fathers and that it needs to be adapted to the local context to make it suitable for men in rural Uganda.

## Background

Globally, an estimated 216 maternal deaths per 100,000 births occurred in 2015 [1]. A large proportion (99%) of these deaths were in low-income countries (LICs) with the African region shouldering almost two thirds (64%) of the global maternal mortality burden [2]. Uganda is a low-income country situated in the eastern part of Africa with an estimated population of 34 million [3]. Uganda’s maternal mortality rate remains high at 368/100,000 live births despite a decline over the past years [4]. The principal direct and indirect causes of maternal morbidity and mortality in LICs often have an underpinning of delayed or little access to maternity care [5]. Birth with a skilled birth attendant (SBA) is central to curbing infections and complications contributing to maternal deaths and morbidity in LICs [6]. A SBA refers to a “trained health worker with midwifery skills such as a midwife, doctor or nurse competent in managing normal pregnancies, and able to appropriately detect and refer complications arising during pregnancy and child birth” [7]. In Uganda, these workers tend to operate within the enabling environment of health facilities, providing pregnant women with safe and clean environments in which to give birth and assistance during the postnatal period [8]. The support pregnant women receive at home and at health facilities impacts on the health outcomes for both mother and baby. Pregnant women often relate to maternity services through a complex social web that reflects power struggles within the kinship and the community [9]. Uptake of health facility birth is still low estimated at 73% in Uganda [4]. Husbands are vital determinants of the likelihood of facility-based delivery by women in LICs in their roles as bread winners, key decision makers in the household and control over economic resources [5]. Involving men in maternal health encourages spousal communication, the making of birth plans and the uptake of health facility based deliveries in a timely manner [10].

Although childbearing and child rearing are regarded as a woman’s role in African communities, decisions around child birth and/or number of children are largely determined by men [11]. Unequal power relations, however pose a problem when men do not support women or actively intervene in health seeking actions [12]. The 1994 International Conference for Population and Development outlined men’s roles in maternal and child health [13]. These roles include men as partners with a responsibility to support women’s health and men as agents of positive change with the ability to change gendered constructions that may impede access to healthcare [14]. Several strategies have been used to encourage male involvement in safe motherhood; group education, mass media campaigns, home visits and games such as soccer and athletics have previously been used as an avenue to reach out to men in communities [12, 15]. Reaching out to men on reproductive health issues is one of the mechanisms by which maternal mortality can be reduced in communities where women’s decision making is limited [16].

Developers of maternal health programmes are increasingly looking for innovative approaches to engage with couples/families and communities to improve health outcomes for pregnant women. Educational board games offer a unique approach to present health information to men, where learning is reinforced through group discussions supporting peer-to-peer interactions [14]. Educational games (board, card and video games) have been designed as a communication tool that bridges the gap between awareness and behaviour change and represent a cost effective mechanism for improving health literacy skills amongst literate and illiterate populations [17]. By providing a visual metaphor, players are given an opportunity to acquire new knowledge through the content of the game, which can enhance critical thinking, collective learning and inter-personal communication in a relaxed environment [18]. Studies on the value of educational board games for health care professionals in high income countries has been published elsewhere [19, 20]. An evaluation of the *Make a Positive Start Today* game reported participants and facilitators’ preferences for a board game over health talks as an education method [17]. Similarly, in a study conducted in Malawi, children and their families were exposed to the *AIDS challenge* game and there was a significant increase in knowledge on HIV/AIDS and change of behaviour [21]. However, the concept of using board games as an educative tool in healthcare is relatively new and evolving in several LICs. Development of educational games for patients and their kin seems especially valuable in cultures where men do not engage much in pregnancy issues and child health care.

## Description of Whose Shoes? Board game

**Fig. 1:**
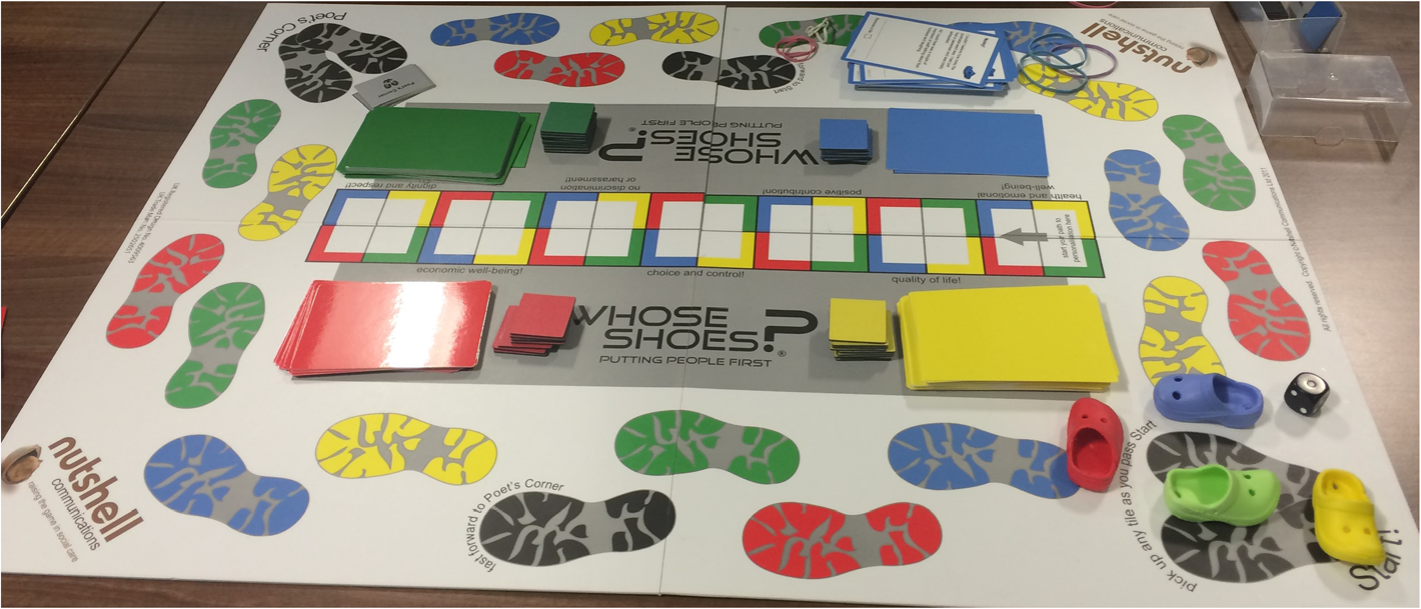
*Whose Shoes?* board game. Photo credit: Gill Phillips

*Whose Shoes?* is the title of the game. This game rests on the premise of empathy, compassion and critical thinking [22]. The *Whose Shoes?* board game consists of: little shoes that act as playing pieces, dice, a board marked with footprints and information cards (Fig. 1). The cards and little shoes are labelled in red, yellow, blue and green colours. An individual player throws the dice, moves a shoe across the board and reads out a card like the colour of shoe.

The game was developed by Gill Phillips as a tool to explore the challenges affecting health and social care in the United Kingdom (UK). The target audience for the workshops have included pregnant women, service users, health professionals involved in delivery of health services and policy makers in the UK [23] (Table 1).

**Table 1:** Content of selected card messages

Diet during pregnancy	Changing the culture	Which place?
• When a pregnant woman has a balanced diet, it helps the proper development of the unborn child and also keeps her from sickness. • A balanced diet includes fruits, vegetables, meat and grain foods.	• My mother told me that she was forced to give birth at home. She lost so much blood that she almost died. • Which cultural norms prevent pregnant women from giving birth in a health facility?	• Women seldom know that they have a right to choose where they wish to give birth. • How can men be of help to their pregnant wives?

Given that men are socialised in groups, peer to peer interactions with fellow men provide a safe environment to explore positive dimensions of their masculine identities. This intervention draws on the health belief model theory with the basic premise that what people know and think affects how they act [24]. An individual’s readiness to act is influenced by perceptions of whether one is susceptible or not to the health problem, how serious the problem is and benefits of avoiding the problem [24]. Evidence from studies on male involvement is largely observational and focussed on motivating men to participate in maternal health [10, 25, 26]. By exploring positive dimensions of their masculine identities and women’s experiences during pregnancy and child birth, men will be granted the ability to reflect and question socially constructed norms that may be detrimental to women’s health. It is hypothesised that once men are given opportunities for learning and self-examination, they will be challenged to embrace more proactive roles in promoting the utilisation of maternity services by women.

## Aim of study

This study sought to pilot the *Whose Shoes?* board game with men of African descent living in the UK. The purpose of the pilot was to first identify whether using a board game to discuss women’s health would be an acceptable intervention, and second to gain feedback on the content of the game and general experiences upon playing the game prior to implementation in Uganda. The essence of conducting pilot studies has been documented elsewhere [27].

## Methods

Using a qualitative approach, men were invited to play the game and participate in a focus group immediately afterwards. Men were purposively sampled to participate in this study. Purposive sampling refers to the deliberate selection of participants based on criteria that would elicit information on a phenomenon of interest [28]. Purposive sampling is a common technique used in qualitative research to select participants in relation to qualities they possess [29]. The inclusion criteria are: men who were fathers and/or married men originally from Africa.

Four men originally from Uganda played *Whose Shoes?* game for 1 h and participated in a focus group discussion (FGD) immediately after the game ended. The FGD lasted for 1 h and was audio recorded with permission from the participants. The researcher (AL) conducted and transcribed the FGD verbatim into English from Luganda (participants chose to speak in local language commonly spoken in Uganda). The game and FGD took place in March 2017. A single FGD was held and considered adequate as it brought sufficient information needed to design relevant messages suitable for the Ugandan audience. The researcher sought to seek views on whether the game was relevant as a health promotional tool and whether messages were appropriate for the Ugandan audience.

FGDs have been documented as an appropriate tool used in studies whose primary aim is to explore attitudes, views and experiences on a research topic in a way that would not be feasible using other methods such as individual interviews [30]. In addition, FGDs can be used at preliminary stages such as pre-test/pilot studies before an intervention is implemented and has been used with a small group of participants [31]. This study chose to use an FGD to elicit responses on men’s views and experiences having played a game to feedback on the design of context specific messages for the Ugandan context. Questions asked included:

- ▪ What aspects of the game did you find most useful in aiding your understanding about topics discussed?
- ▪ Were there any parts that were unclear/not relevant?
- ▪ What benefits did you experience upon engaging with the game?
- ▪ What did you like/dislike about the game?
- ▪ Is there anything you would have changed about the game?

## Recruitment procedure

Men were recruited through a local church in East London. The church was used as a recruitment platform because religion constitutes an important medium through which Africans in the diaspora construct their identity and cultivate a sense of belonging [32]. African immigrants including men, irrespective of their religious beliefs readily attend events organised by the church as a social network. Information about the study was presented to people in attendance through ‘announcements’ and further spread through word of mouth to individuals not in attendance by the researcher. All men who expressed interest in the study met with the researcher after the event at church and more information was provided accordingly.

A participant information sheet was given to men to consider participation 1 week prior to the study. It was emphasised that participation was voluntary and participants given the opportunity to ask questions. Participants signed a participant agreement form before the commencement of the study.

## Data analysis

Data for this study were analysed using template analysis (TA) [33]. This is a technique used in thematic analysis to organise and analyse textual data [34, 35]. An initial outline template is applied to the data and revised to incorporate new emergent themes in a hierarchical structure. It follows an iterative process which may involve adding, collapsing and deletion of codes/themes in the outline template, until a final template representing all the data is developed [33].

This method of analysis was selected because: TA can be used to address a wide range of research questions including people’s experiences and perspectives [33]. This study explored men’s experiences of engaging with the game and perceptions on the use of games as a learning tool in maternity services. It provides a researcher with an opportunity to develop priori codes and themes more extensively where the richest data are found in relation to the research question [35]. Also, the flexibility of the coding structure allows researchers to explore the relevant aspects of the data in real depth. As this was a pilot study, the researchers were keen on receiving feedback on the content of the game that would be used to design context specific messages for men in Uganda.

## Analysis procedure

The discipline of producing a template enables the researcher to take a systematic and well-structured approach to data handling. The use of an initial template followed by the iterative process of coding means that the method facilitates careful consideration of how codes/themes are defined and ensures rigour. The transcript was read several times to become familiar with the data and then annotated with emerging codes which were added to the template. An initial coding template following the structure of the FGD guide was developed. The template was revised throughout this process with additional themes and sub themes inserted, deleted or collapsed under a new heading as the analysis progressed until the final template was developed. For confidentiality purposes, pseudonyms were used and access to data was restricted to only the researchers involved in the study. Trustworthiness was enhanced through the use of template analysis method; returning to the data repeatedly to check for accuracy in interpretation; dialogue with two senior researchers with extensive experience in maternal health and qualitative research.

## Results

The analysis of the focus group discussion generated four themes or key issues: 1) aspects of the game that aided men’s understanding on topics discussed (learning aids and messages); 2) benefits of the game (empathy and change in perspective); 3) general attitudes about the game; and 4) recommendations for the game (context specific messages and should be used in the rural setting).

### Aspects of the game that aided men’s understanding

#### Learning aids used

##### Little shoes

Participants spoke about the symbols that were visual such as dice, little croc shoes, and pathways clearly marked on the board game.

*The shoe is a core point, it has ignited my thoughts to stand in the shoes of women, to walk in her shoes.* P4

*The significance of the shoe as my colleague has emphasised is that for many of us here even in daily life situations, we do not place ourselves in the shoes of others…*P1

Using the symbol of a shoe enabled participants to reflect on women’s experiences in pregnancy and roles at home. Participants appeared fascinated by the concept of the little shoes and throwing the dice on the board. This captured men’s attention and inspired them to pause and examine their actions through a woman’s lens.

#### Messages

##### Poem

Some of the participants were motivated through the choice of words used to relay messages to men in form of poems, questions and statements.

*When we were playing the game, the dice I threw, took me to the poet’s corner and the poem I ended up picking said let us work together, they used the phrase of real people and not roles.* P2

*It has opened my understanding in that, although we talk about it, we sometimes overlook it but the way the questions were phrased…*P4

Messages reinforced a collective effort in addressing women’s health concerns. It reinforced the need for men to view themselves as part of the facilitaters of change.

##### Use of real life events & experiences

All participants appreciated the use of real life situations on topics discussed which inspired them to share their own experiences whilst engaging with health professionals and family life.

*We are here talking about real life scenarios happening in Kitgum, Soroti, Masaka [districts in Uganda]. It is real scenarios, no doctor, no medicine, a woman is pregnant, vulnerable, she has to give birth. I look at the fact that all this is true.* P2

The messages on the cards talked about experiences women go through such as dignity in care, nutrition, birth preparedness, male involvement among others. These were experiences the men in the group could relate to being fathers in stable relationships.

### Benefits of the game

#### Empathy

Participants placed emphasis on the concept of ‘little shoe’ which draws men’s attention to think about women’s experiences during pregnancy and child birth

*This particular game brings men’s attention to not just overlook women’s issues especially pregnant women but to go deeper to think about it as several men father children, take them to school but in his thoughts, he has never really stepped into the shoes of women.* P3

#### Enhances change in perspective

Reflection appears to act as a mirror where men view their actions through a woman’s lens and are able to weigh in on their actions.

*I think for me what I have picked from this game which is most important is about perspective and mind set.* P1

The game provided a platform to discuss women’s experiences with fellow men. Such opportunities for self-examination and critical thinking enhances a change in mind set about cultural roles accrued to men and women by society.

### General attitudes about the game

All men in this study were receptive towards the use of board games to engage with men in maternity services.

*Yes, you chose the best [game], I think we need to encourage all men everywhere especially in Uganda or Africa to stand in the shoes of women.* P2

Participants were keen on the concept of *Whose Shoes*, drawing on empathy to reflect on experiences women face during pregnancy and child birth. This was also reflected in the roles women perform in the home beyond child bearing such as care giving and house work.

#### Environment

Despite the fact that the men in this study generally liked the concept of *Whose Shoes?* game, there appeared mixed reactions with regards to the relevancy of the game to men living in the UK.

*let us say that almost you are preaching to the converted, since we have lived in this country and also been here for long, we already got a perspective irrespective of what we had that a man does not do this and that, now we all do things differently.* P1

*…even us here, it has helped us except that most of us here can relate on the same level because we know, we’ve been there, we do it and the environment we live in, is normal [for men to be involved].* P2

Some of the participants were inspired to reflect on their involvement in maternity services, whilst others were already actively involved in offering support including chores around the home.

Participants highlighted different perspectives between men who are exposed and unexposed to male involvement due to contextual factors such as environment.

*You will find that generally we who are here [UK], the perspective we have will be different from the everyday Ugandan man because of the different experiences and environment.* P4

Men in this study appeared to have adopted a new perspective of being involved in women’s health as a result of living in the UK. Exposure to a way of life that is different from the Ugandan context had influenced men’s decision to be involved.

#### Easy to understand and use

In this study, men found the components of the game and messages easy to understand and could easily relate with the content of the game since they were parents.

*This shoe whether you went to school or not, whether whoever assisted with the delivery did that in a health facility or not, I want a man to wear the shoes of this woman…* P2

The use of symbols like little shoes and footprints on the board are visual tools that drew men’s attention to reflect on women’s experiences. The simplicity of the game implies that it can be used with men across different socio-economic status and education levels.

### Recommendations for the game

#### Context

Participants emphasised the need for the game to be applied to the Ugandan context to make it is more relevant so that it has the potential to make a unique contribution

*If you shift it to Uganda, for so many men, it would be something new. Pregnancy is hers to deal with and as a man, I come in to boast about being a father to the child and that is it. There is a great need for change to come to our communities.* P3

*If you went deep in the villages like in Mukono* [district], *all your trying to aim for is a change in perspective and mind set… it would be more eye opening to them because you will find that for them when a woman gives birth, I know about it when she comes back with a baby or when am picking her up from the hospital. That is where his role starts as a man.* P1

Considerable importance was placed on engaging men in the rural setting especially the villages where cultural values are dominant and social norms dictate men and women’s roles.

#### Messages

Participants proposed the need to design messages that are not only context specific but compelling messages that could provide men with opportunities for self-reflection.

*The messages must be hard hitting and relevant in that it has to give people food for thought like when a man says traditionally, we have always done things this way, the questions become tailored to that.* P3

*As a concept [game] it is very good but then it has to be more hard hitting that if I place someone in that position, it gives them a chance to think. A woman does not complain but she suffers in silence but it does not remove the fact that a man needs to change.* P1

Participants highlighted the need for messages addressing socio cultural norms that hinder male participation in maternity services and women’s access to health services.

In addition, messages could reinforce men’s active involvement during pregnancy and support each other as a team

*If we work on changing our mind sets as men especially in Uganda that when a woman is pregnant, from that day we wear the same shoe and it becomes my responsibility as well to put on a woman’s shoes, know her experiences and support one another as we are in this together.* P2

Participants suggested the inclusion of messages with an emphasis on pregnancy and child birth viewed as shared responsibility amongst couples.

## Discussion

Overall, men were receptive about the use of a game and embraced the concept of using real life scenarios. This reflects the findings of an evaluation study conducted in Uganda on *Make a Positive Start Today* game, which reported participants’ preference for the use of a board game compared to health talks as an education method [17]. Men in this study were particularly captivated by the visual aids used such as little shoes, throwing dice and footprints on a board. A visual display of the game reiterated the importance of walking in women’s shoes. The role of board games in motivating players has been documented elsewhere [19]. In the *Clinical Pharmacology Game*, medical students stated having enjoyed playing the game which involved rolling the dice and moving patient characters on a board [20].

For the pilot study, a subset of cards was chosen from the package of *Whose Shoes?* cards and some new cards were added that were not part of the original UK package. The content of the cards included messages on dignity in care, team work, empathy, men’s roles and birth preparedness and complication readiness. These cards were considered most relevant to the subject of pregnancy and childbirth and appropriate for the Ugandan audience. Men reported having understood the messages on the cards, which drew rich discussions on the topics of interest and those beyond the scope of the study. According to Okitika and colleagues [36] interactive games can increase interest in global health issues among the lay public especially among young people. Similarly, others observed that educational games can be used as a learning aid to promote health literacy among individuals with low literacy levels [37].

Piloting the *Whose Shoes?* board game with Ugandan men provided valuable insight into practical issues such as whether board games are acceptable and appropriate as a mechanism to facilitate change in behaviour. The findings also reiterated the importance of adapting the game to suit the local context. This included ensuring that cards and data collection tools were translated into Luganda, the local language of the population in the main study, and that male facilitators were on hand to work with male and female researchers and women during the intervention and interviews respectively. Smith and colleagues [38] highlighted the need to pilot studies to test selection and/recruitment processes, data collection instruments, and the duration and quality measures on a relatively small scale before the main study is carried out.

## Limitations of the study

This pilot study consisted of a focus group discussion of four Ugandan men. Whislt the participants still maintained regular contact/visits to their villages in Uganda, the authors acknowledge the likelihood of a change in perception and behaviour having been exposured to life in the UK. The limitation of using template analysis as an approach in analysing qualitative data is the risk of losing sight of the original research aims and the focus on the existing template as an end result rather than a means to achieving an outcome. Although considered a minimal limitation in this particular study the researcher acknowledges the fact that all approaches to qualitative analysis are not without flaws and as therefore addressed this concern through steering clear of the reasons for adopting a template as a process of understanding the data and not the purpose of the analysis.

## Conclusion

This study provides preliminary data on the relevancy and efficacy of using board games in maternal health. Key messages from the focus group appeared to be that the board game is more than acceptable to fathers and that it needs to be adapted to the local context to make it suitable for men in rural Uganda. The main study, using a before and after design, will assess the feasibility of implementing a board game with men to encourage uptake of health facility births by women in Uganda.

## Abbreviations

LIC: Low income country
SBA: Skilled birth attendant
TA: Template analysis
UK: United Kingdom
